# Supplementary material for: Modeling glioblastoma heterogeneity as a dynamic network of cell states
Source: Mol Syst Biol. 2021 Sep 16;17(9):e10105. doi: 10.15252/msb.202010105 (PMC8444284; doi:10.15252/msb.202010105)
Supplement: Supplementary file 5 — Source Data for Figure 3 [file MSB-17-e10105-s001.zip › Figure3A_sourcedata/GSEA_3065/nosigdb_state5.GseaPreranked.1622718435914/VANGRONINGEN_MES.html]

Details for gene set VANGRONINGEN\_MES[GSEA]

|  || Dataset | state5 |
| Phenotype | NoPhenotypeAvailable |
| Upregulated in class | na\_pos |
| GeneSet | VANGRONINGEN\_MES |
| Enrichment Score (ES) | 0.44936907 |
| Normalized Enrichment Score (NES) | 2.1464846 |
| Nominal p-value | 0.0 |
| FDR q-value | 2.7857145E-4 |
| FWER p-Value | 0.001 |
Table: GSEA Results Summary

  

Fig 1: Enrichment plot: VANGRONINGEN\_MES      
 Profile of the Running ES Score & Positions of GeneSet Members on the Rank Ordered List

  

| PROBE | GENE SYMBOL | GENE\_TITLE | RANK IN GENE LIST | RANK METRIC SCORE | RUNNING ES | CORE ENRICHMENT || 1 | PTN |  |  | 19 | 0.824 | 0.0078 | Yes |
| 2 | CD63 |  |  | 24 | 0.769 | 0.0167 | Yes |
| 3 | PDIA6 |  |  | 29 | 0.742 | 0.0253 | Yes |
| 4 | HSP90B1 |  |  | 33 | 0.729 | 0.0338 | Yes |
| 5 | SCRG1 |  |  | 36 | 0.715 | 0.0422 | Yes |
| 6 | APP |  |  | 38 | 0.712 | 0.0508 | Yes |
| 7 | HSPA5 |  |  | 43 | 0.698 | 0.0588 | Yes |
| 8 | TNC |  |  | 45 | 0.680 | 0.0669 | Yes |
| 9 | PDIA3 |  |  | 49 | 0.652 | 0.0745 | Yes |
| 10 | LAPTM4A |  |  | 51 | 0.641 | 0.0822 | Yes |
| 11 | COL4A1 |  |  | 54 | 0.634 | 0.0897 | Yes |
| 12 | B2M |  |  | 61 | 0.601 | 0.0963 | Yes |
| 13 | DDOST |  |  | 67 | 0.586 | 0.1028 | Yes |
| 14 | CALU |  |  | 78 | 0.563 | 0.1085 | Yes |
| 15 | PRDX4 |  |  | 80 | 0.553 | 0.1151 | Yes |
| 16 | PON2 |  |  | 84 | 0.546 | 0.1214 | Yes |
| 17 | COL4A2 |  |  | 93 | 0.540 | 0.1270 | Yes |
| 18 | LMAN1 |  |  | 95 | 0.533 | 0.1334 | Yes |
| 19 | PRCP |  |  | 103 | 0.523 | 0.1389 | Yes |
| 20 | PLEKHH2 |  |  | 106 | 0.520 | 0.1450 | Yes |
| 21 | TIMP1 |  |  | 109 | 0.518 | 0.1511 | Yes |
| 22 | PDIA4 |  |  | 112 | 0.514 | 0.1571 | Yes |
| 23 | WLS |  |  | 116 | 0.509 | 0.1629 | Yes |
| 24 | ITM2B |  |  | 117 | 0.509 | 0.1691 | Yes |
| 25 | ITGAV |  |  | 118 | 0.508 | 0.1753 | Yes |
| 26 | TMED9 |  |  | 119 | 0.508 | 0.1815 | Yes |
| 27 | MANF |  |  | 130 | 0.499 | 0.1864 | Yes |
| 28 | RRBP1 |  |  | 147 | 0.486 | 0.1904 | Yes |
| 29 | FAT1 |  |  | 155 | 0.478 | 0.1954 | Yes |
| 30 | PPIB |  |  | 156 | 0.477 | 0.2012 | Yes |
| 31 | ITGB1 |  |  | 157 | 0.477 | 0.2070 | Yes |
| 32 | TMBIM4 |  |  | 181 | 0.457 | 0.2099 | Yes |
| 33 | SPCS3 |  |  | 183 | 0.454 | 0.2153 | Yes |
| 34 | DNAJC3 |  |  | 192 | 0.444 | 0.2198 | Yes |
| 35 | GDF15 |  |  | 209 | 0.433 | 0.2232 | Yes |
| 36 | RCN1 |  |  | 217 | 0.427 | 0.2276 | Yes |
| 37 | MAML2 |  |  | 220 | 0.425 | 0.2325 | Yes |
| 38 | TM9SF2 |  |  | 229 | 0.419 | 0.2367 | Yes |
| 39 | SYPL1 |  |  | 231 | 0.418 | 0.2416 | Yes |
| 40 | SERPINE2 |  |  | 238 | 0.414 | 0.2460 | Yes |
| 41 | PTPRG |  |  | 245 | 0.411 | 0.2503 | Yes |
| 42 | EPHA3 |  |  | 246 | 0.411 | 0.2553 | Yes |
| 43 | EMP1 |  |  | 258 | 0.404 | 0.2589 | Yes |
| 44 | PLOD3 |  |  | 270 | 0.398 | 0.2625 | Yes |
| 45 | JAM3 |  |  | 276 | 0.395 | 0.2667 | Yes |
| 46 | ROBO1 |  |  | 277 | 0.395 | 0.2715 | Yes |
| 47 | CD164 |  |  | 279 | 0.394 | 0.2762 | Yes |
| 48 | LAMP1 |  |  | 304 | 0.380 | 0.2780 | Yes |
| 49 | POSTN |  |  | 316 | 0.375 | 0.2813 | Yes |
| 50 | SURF4 |  |  | 326 | 0.370 | 0.2848 | Yes |
| 51 | SPARC |  |  | 327 | 0.370 | 0.2893 | Yes |
| 52 | TMEM50A |  |  | 341 | 0.364 | 0.2922 | Yes |
| 53 | CTSC |  |  | 347 | 0.362 | 0.2960 | Yes |
| 54 | CD59 |  |  | 354 | 0.360 | 0.2997 | Yes |
| 55 | CD44 |  |  | 378 | 0.349 | 0.3012 | Yes |
| 56 | LAMC1 |  |  | 382 | 0.348 | 0.3051 | Yes |
| 57 | KDELR2 |  |  | 391 | 0.345 | 0.3084 | Yes |
| 58 | VCL |  |  | 400 | 0.342 | 0.3116 | Yes |
| 59 | COL5A2 |  |  | 407 | 0.337 | 0.3150 | Yes |
| 60 | ASPH |  |  | 413 | 0.335 | 0.3185 | Yes |
| 61 | PXDN |  |  | 415 | 0.335 | 0.3225 | Yes |
| 62 | ADAM9 |  |  | 421 | 0.334 | 0.3260 | Yes |
| 63 | GPC6 |  |  | 443 | 0.326 | 0.3275 | Yes |
| 64 | ATP2B4 |  |  | 445 | 0.326 | 0.3313 | Yes |
| 65 | SERPINH1 |  |  | 460 | 0.322 | 0.3336 | Yes |
| 66 | RNFT1 |  |  | 463 | 0.321 | 0.3373 | Yes |
| 67 | SLC38A2 |  |  | 472 | 0.319 | 0.3403 | Yes |
| 68 | DNAJC1 |  |  | 517 | 0.306 | 0.3389 | Yes |
| 69 | TXNDC12 |  |  | 527 | 0.304 | 0.3415 | Yes |
| 70 | TRAM1 |  |  | 528 | 0.304 | 0.3452 | Yes |
| 71 | COL6A1 |  |  | 536 | 0.301 | 0.3481 | Yes |
| 72 | FAM3C |  |  | 538 | 0.301 | 0.3516 | Yes |
| 73 | MRC2 |  |  | 543 | 0.299 | 0.3548 | Yes |
| 74 | FGFR1 |  |  | 545 | 0.299 | 0.3583 | Yes |
| 75 | ATP1B1 |  |  | 548 | 0.298 | 0.3617 | Yes |
| 76 | ITM2C |  |  | 551 | 0.297 | 0.3651 | Yes |
| 77 | DNAJC10 |  |  | 564 | 0.294 | 0.3673 | Yes |
| 78 | F2R |  |  | 568 | 0.293 | 0.3705 | Yes |
| 79 | SSR3 |  |  | 575 | 0.291 | 0.3733 | Yes |
| 80 | PLOD2 |  |  | 613 | 0.282 | 0.3724 | Yes |
| 81 | TNFRSF12A |  |  | 615 | 0.282 | 0.3758 | Yes |
| 82 | LAMB1 |  |  | 619 | 0.281 | 0.3788 | Yes |
| 83 | GRN |  |  | 626 | 0.279 | 0.3815 | Yes |
| 84 | IL6ST |  |  | 627 | 0.279 | 0.3849 | Yes |
| 85 | HEXB |  |  | 628 | 0.279 | 0.3883 | Yes |
| 86 | CREB3L2 |  |  | 629 | 0.279 | 0.3917 | Yes |
| 87 | CDH11 |  |  | 630 | 0.279 | 0.3951 | Yes |
| 88 | KDELC2 |  |  | 631 | 0.279 | 0.3985 | Yes |
| 89 | HTRA1 |  |  | 690 | 0.266 | 0.3950 | Yes |
| 90 | TRAM2 |  |  | 693 | 0.265 | 0.3980 | Yes |
| 91 | IGF2R |  |  | 705 | 0.260 | 0.3999 | Yes |
| 92 | CRELD2 |  |  | 718 | 0.258 | 0.4016 | Yes |
| 93 | PPIC |  |  | 723 | 0.257 | 0.4043 | Yes |
| 94 | PPT1 |  |  | 744 | 0.251 | 0.4050 | Yes |
| 95 | NOTCH2 |  |  | 756 | 0.249 | 0.4067 | Yes |
| 96 | DPY19L1 |  |  | 775 | 0.246 | 0.4077 | Yes |
| 97 | PHTF2 |  |  | 790 | 0.243 | 0.4090 | Yes |
| 98 | P4HA1 |  |  | 792 | 0.242 | 0.4118 | Yes |
| 99 | STAT3 |  |  | 797 | 0.242 | 0.4143 | Yes |
| 100 | HYOU1 |  |  | 818 | 0.238 | 0.4149 | Yes |
| 101 | EXTL2 |  |  | 821 | 0.237 | 0.4175 | Yes |
| 102 | AEBP1 |  |  | 825 | 0.237 | 0.4200 | Yes |
| 103 | FUCA2 |  |  | 862 | 0.230 | 0.4187 | Yes |
| 104 | GPX8 |  |  | 874 | 0.229 | 0.4202 | Yes |
| 105 | PTPRK |  |  | 887 | 0.228 | 0.4215 | Yes |
| 106 | MYDGF |  |  | 900 | 0.226 | 0.4229 | Yes |
| 107 | CTSB |  |  | 902 | 0.225 | 0.4255 | Yes |
| 108 | SSR1 |  |  | 922 | 0.221 | 0.4260 | Yes |
| 109 | OSTC |  |  | 939 | 0.220 | 0.4268 | Yes |
| 110 | ATP2B1 |  |  | 953 | 0.218 | 0.4280 | Yes |
| 111 | MAGT1 |  |  | 1006 | 0.210 | 0.4245 | Yes |
| 112 | SLC16A4 |  |  | 1007 | 0.210 | 0.4270 | Yes |
| 113 | TWSG1 |  |  | 1016 | 0.208 | 0.4286 | Yes |
| 114 | FKBP14 |  |  | 1019 | 0.208 | 0.4309 | Yes |
| 115 | GALNT10 |  |  | 1024 | 0.208 | 0.4330 | Yes |
| 116 | DKK3 |  |  | 1050 | 0.204 | 0.4326 | Yes |
| 117 | ATXN1 |  |  | 1062 | 0.203 | 0.4338 | Yes |
| 118 | LEPROT |  |  | 1072 | 0.202 | 0.4352 | Yes |
| 119 | FSTL1 |  |  | 1086 | 0.200 | 0.4361 | Yes |
| 120 | SLC35F5 |  |  | 1093 | 0.199 | 0.4378 | Yes |
| 121 | NPTN |  |  | 1102 | 0.198 | 0.4393 | Yes |
| 122 | BOC |  |  | 1113 | 0.197 | 0.4405 | Yes |
| 123 | QSOX1 |  |  | 1125 | 0.195 | 0.4416 | Yes |
| 124 | ADAM19 |  |  | 1173 | 0.188 | 0.4384 | Yes |
| 125 | COPA |  |  | 1189 | 0.186 | 0.4390 | Yes |
| 126 | SDC4 |  |  | 1193 | 0.186 | 0.4409 | Yes |
| 127 | SDF4 |  |  | 1242 | 0.180 | 0.4375 | Yes |
| 128 | CMTM6 |  |  | 1244 | 0.180 | 0.4396 | Yes |
| 129 | ADGRG6 |  |  | 1259 | 0.178 | 0.4401 | Yes |
| 130 | TFPI |  |  | 1278 | 0.175 | 0.4401 | Yes |
| 131 | MBTPS1 |  |  | 1312 | 0.170 | 0.4384 | Yes |
| 132 | COL5A1 |  |  | 1328 | 0.168 | 0.4387 | Yes |
| 133 | IFITM3 |  |  | 1332 | 0.168 | 0.4404 | Yes |
| 134 | SLC39A14 |  |  | 1340 | 0.167 | 0.4416 | Yes |
| 135 | FN1 |  |  | 1342 | 0.167 | 0.4435 | Yes |
| 136 | MYADM |  |  | 1345 | 0.167 | 0.4453 | Yes |
| 137 | ANTXR1 |  |  | 1372 | 0.164 | 0.4443 | Yes |
| 138 | ERLIN1 |  |  | 1373 | 0.164 | 0.4463 | Yes |
| 139 | TNFRSF1A |  |  | 1377 | 0.163 | 0.4479 | Yes |
| 140 | GJA1 |  |  | 1399 | 0.162 | 0.4474 | Yes |
| 141 | SRPX |  |  | 1409 | 0.161 | 0.4484 | Yes |
| 142 | SYNJ2 |  |  | 1418 | 0.160 | 0.4494 | Yes |
| 143 | LPP |  |  | 1489 | 0.154 | 0.4431 | No |
| 144 | SLC30A7 |  |  | 1518 | 0.151 | 0.4417 | No |
| 145 | LIFR |  |  | 1520 | 0.150 | 0.4434 | No |
| 146 | CRISPLD1 |  |  | 1545 | 0.147 | 0.4424 | No |
| 147 | TMEFF2 |  |  | 1546 | 0.147 | 0.4442 | No |
| 148 | EGFR |  |  | 1547 | 0.147 | 0.4460 | No |
| 149 | GNS |  |  | 1591 | 0.142 | 0.4427 | No |
| 150 | CYBRD1 |  |  | 1607 | 0.141 | 0.4427 | No |
| 151 | CKAP4 |  |  | 1636 | 0.139 | 0.4411 | No |
| 152 | SPARCL1 |  |  | 1663 | 0.136 | 0.4398 | No |
| 153 | NPC2 |  |  | 1667 | 0.136 | 0.4411 | No |
| 154 | FZD2 |  |  | 1701 | 0.133 | 0.4388 | No |
| 155 | ADGRE5 |  |  | 1730 | 0.131 | 0.4372 | No |
| 156 | FBN1 |  |  | 1756 | 0.129 | 0.4359 | No |
| 157 | PDGFC |  |  | 1789 | 0.127 | 0.4337 | No |
| 158 | MMP2 |  |  | 1824 | 0.125 | 0.4313 | No |
| 159 | CYR61 |  |  | 1850 | 0.123 | 0.4299 | No |
| 160 | LRRC17 |  |  | 1859 | 0.122 | 0.4304 | No |
| 161 | SEMA3C |  |  | 1870 | 0.121 | 0.4307 | No |
| 162 | ATP10D |  |  | 1898 | 0.119 | 0.4290 | No |
| 163 | SFT2D2 |  |  | 1911 | 0.118 | 0.4291 | No |
| 164 | CCDC80 |  |  | 1921 | 0.117 | 0.4294 | No |
| 165 | MAN2A1 |  |  | 1922 | 0.117 | 0.4309 | No |
| 166 | KDELR3 |  |  | 1926 | 0.117 | 0.4319 | No |
| 167 | DDR2 |  |  | 2000 | 0.112 | 0.4248 | No |
| 168 | IL13RA1 |  |  | 2016 | 0.110 | 0.4244 | No |
| 169 | LOXL2 |  |  | 2028 | 0.110 | 0.4245 | No |
| 170 | PLPP1 |  |  | 2048 | 0.108 | 0.4236 | No |
| 171 | MEST |  |  | 2059 | 0.108 | 0.4237 | No |
| 172 | OLFML2A |  |  | 2094 | 0.106 | 0.4211 | No |
| 173 | ERBIN |  |  | 2101 | 0.105 | 0.4216 | No |
| 174 | SCPEP1 |  |  | 2106 | 0.105 | 0.4225 | No |
| 175 | CXCL12 |  |  | 2165 | 0.101 | 0.4169 | No |
| 176 | TSPAN4 |  |  | 2226 | 0.096 | 0.4112 | No |
| 177 | EDEM1 |  |  | 2295 | 0.092 | 0.4044 | No |
| 178 | HES1 |  |  | 2302 | 0.092 | 0.4048 | No |
| 179 | EGR1 |  |  | 2336 | 0.090 | 0.4021 | No |
| 180 | PTGFRN |  |  | 2339 | 0.089 | 0.4029 | No |
| 181 | MGAT2 |  |  | 2365 | 0.088 | 0.4011 | No |
| 182 | MBNL1 |  |  | 2373 | 0.088 | 0.4013 | No |
| 183 | NES |  |  | 2417 | 0.084 | 0.3974 | No |
| 184 | FZD7 |  |  | 2420 | 0.084 | 0.3981 | No |
| 185 | RECK |  |  | 2431 | 0.083 | 0.3980 | No |
| 186 | LIPA |  |  | 2451 | 0.082 | 0.3968 | No |
| 187 | CRTAP |  |  | 2472 | 0.081 | 0.3955 | No |
| 188 | ARHGEF40 |  |  | 2547 | 0.077 | 0.3878 | No |
| 189 | EFEMP2 |  |  | 2557 | 0.076 | 0.3877 | No |
| 190 | FIBIN |  |  | 2599 | 0.074 | 0.3838 | No |
| 191 | COL6A2 |  |  | 2660 | 0.071 | 0.3777 | No |
| 192 | HS3ST3A1 |  |  | 2678 | 0.069 | 0.3766 | No |
| 193 | PTBP1 |  |  | 2754 | 0.065 | 0.3687 | No |
| 194 | HIPK3 |  |  | 2781 | 0.064 | 0.3664 | No |
| 195 | LRP10 |  |  | 2782 | 0.064 | 0.3672 | No |
| 196 | GPR137B |  |  | 2815 | 0.062 | 0.3642 | No |
| 197 | AMMECR1 |  |  | 2897 | 0.058 | 0.3555 | No |
| 198 | DMD |  |  | 2914 | 0.057 | 0.3543 | No |
| 199 | WNT5A |  |  | 2927 | 0.056 | 0.3536 | No |
| 200 | PEAK1 |  |  | 2939 | 0.056 | 0.3530 | No |
| 201 | COL11A1 |  |  | 2950 | 0.055 | 0.3526 | No |
| 202 | SEL1L3 |  |  | 2958 | 0.055 | 0.3524 | No |
| 203 | TM4SF1 |  |  | 3045 | 0.050 | 0.3430 | No |
| 204 | INSIG1 |  |  | 3055 | 0.049 | 0.3426 | No |
| 205 | NID1 |  |  | 3069 | 0.048 | 0.3417 | No |
| 206 | CBFB |  |  | 3166 | 0.043 | 0.3310 | No |
| 207 | SFT2D1 |  |  | 3198 | 0.041 | 0.3279 | No |
| 208 | IGFBP5 |  |  | 3230 | 0.040 | 0.3248 | No |
| 209 | ELAVL1 |  |  | 3237 | 0.039 | 0.3246 | No |
| 210 | SKIL |  |  | 3291 | 0.037 | 0.3189 | No |
| 211 | S1PR3 |  |  | 3308 | 0.036 | 0.3175 | No |
| 212 | TOR1AIP1 |  |  | 3313 | 0.036 | 0.3174 | No |
| 213 | TCF7L2 |  |  | 3356 | 0.033 | 0.3130 | No |
| 214 | KLF6 |  |  | 3447 | 0.029 | 0.3029 | No |
| 215 | ELK4 |  |  | 3506 | 0.027 | 0.2965 | No |
| 216 | CYFIP1 |  |  | 3515 | 0.026 | 0.2959 | No |
| 217 | ACAP2 |  |  | 3549 | 0.025 | 0.2923 | No |
| 218 | FNDC3B |  |  | 3561 | 0.025 | 0.2914 | No |
| 219 | SNAP23 |  |  | 3621 | 0.023 | 0.2848 | No |
| 220 | CTSO |  |  | 3624 | 0.023 | 0.2848 | No |
| 221 | TSC22D2 |  |  | 3647 | 0.022 | 0.2825 | No |
| 222 | WIPI1 |  |  | 3722 | 0.019 | 0.2742 | No |
| 223 | NRP1 |  |  | 3735 | 0.019 | 0.2730 | No |
| 224 | THBS1 |  |  | 3755 | 0.017 | 0.2710 | No |
| 225 | MEOX2 |  |  | 3785 | 0.016 | 0.2679 | No |
| 226 | SPRY4 |  |  | 3821 | 0.015 | 0.2640 | No |
| 227 | STK38L |  |  | 3838 | 0.014 | 0.2623 | No |
| 228 | GAS1 |  |  | 3841 | 0.014 | 0.2622 | No |
| 229 | ARMCX2 |  |  | 3899 | 0.012 | 0.2557 | No |
| 230 | SMAD3 |  |  | 3915 | 0.011 | 0.2541 | No |
| 231 | RHOJ |  |  | 3980 | 0.008 | 0.2468 | No |
| 232 | RGL1 |  |  | 4007 | 0.007 | 0.2439 | No |
| 233 | TRIM5 |  |  | 4008 | 0.007 | 0.2439 | No |
| 234 | HOMER1 |  |  | 4062 | 0.005 | 0.2378 | No |
| 235 | C6orf120 |  |  | 4065 | 0.004 | 0.2377 | No |
| 236 | IFI16 |  |  | 4066 | 0.004 | 0.2377 | No |
| 237 | TPBG |  |  | 4075 | 0.004 | 0.2368 | No |
| 238 | SPRED1 |  |  | 4106 | 0.003 | 0.2334 | No |
| 239 | ITPRIPL2 |  |  | 4156 | 0.001 | 0.2277 | No |
| 240 | DSE |  |  | 4414 | -0.011 | 0.1980 | No |
| 241 | GORAB |  |  | 4425 | -0.011 | 0.1970 | No |
| 242 | SPATA20 |  |  | 4465 | -0.013 | 0.1926 | No |
| 243 | L3HYPDH |  |  | 4494 | -0.014 | 0.1895 | No |
| 244 | SIX1 |  |  | 4571 | -0.017 | 0.1809 | No |
| 245 | SDC2 |  |  | 4596 | -0.018 | 0.1783 | No |
| 246 | PLEKHA2 |  |  | 4629 | -0.019 | 0.1748 | No |
| 247 | GNAI1 |  |  | 4664 | -0.021 | 0.1711 | No |
| 248 | REST |  |  | 4679 | -0.021 | 0.1698 | No |
| 249 | CALD1 |  |  | 4714 | -0.022 | 0.1661 | No |
| 250 | LUZP1 |  |  | 4767 | -0.025 | 0.1604 | No |
| 251 | TSC22D3 |  |  | 4775 | -0.025 | 0.1598 | No |
| 252 | SPRY1 |  |  | 4844 | -0.027 | 0.1523 | No |
| 253 | ZNF217 |  |  | 4859 | -0.028 | 0.1510 | No |
| 254 | SVIL |  |  | 4953 | -0.031 | 0.1406 | No |
| 255 | PLSCR1 |  |  | 5043 | -0.034 | 0.1307 | No |
| 256 | IFITM2 |  |  | 5072 | -0.036 | 0.1278 | No |
| 257 | FILIP1L |  |  | 5096 | -0.037 | 0.1256 | No |
| 258 | TGFBR2 |  |  | 5140 | -0.038 | 0.1211 | No |
| 259 | KIF13A |  |  | 5331 | -0.046 | 0.0996 | No |
| 260 | SSBP4 |  |  | 5438 | -0.050 | 0.0879 | No |
| 261 | KLF10 |  |  | 5448 | -0.050 | 0.0874 | No |
| 262 | ERRFI1 |  |  | 5451 | -0.050 | 0.0878 | No |
| 263 | FAM129A |  |  | 5477 | -0.052 | 0.0855 | No |
| 264 | OGFRL1 |  |  | 5490 | -0.052 | 0.0848 | No |
| 265 | PHLDB2 |  |  | 5520 | -0.054 | 0.0821 | No |
| 266 | ELF1 |  |  | 5530 | -0.054 | 0.0817 | No |
| 267 | ARHGAP1 |  |  | 5533 | -0.054 | 0.0821 | No |
| 268 | YAP1 |  |  | 5575 | -0.056 | 0.0780 | No |
| 269 | TPM2 |  |  | 5660 | -0.060 | 0.0690 | No |
| 270 | SUCLG2 |  |  | 5712 | -0.062 | 0.0639 | No |
| 271 | QKI |  |  | 5715 | -0.062 | 0.0644 | No |
| 272 | TFE3 |  |  | 5801 | -0.065 | 0.0553 | No |
| 273 | RIT1 |  |  | 5824 | -0.066 | 0.0536 | No |
| 274 | SHC1 |  |  | 5833 | -0.067 | 0.0534 | No |
| 275 | SASH1 |  |  | 5850 | -0.067 | 0.0524 | No |
| 276 | RAB29 |  |  | 5917 | -0.070 | 0.0456 | No |
| 277 | KDM5B |  |  | 5937 | -0.071 | 0.0443 | No |
| 278 | SEPT10 |  |  | 5944 | -0.072 | 0.0444 | No |
| 279 | ACADVL |  |  | 6028 | -0.076 | 0.0357 | No |
| 280 | STAT1 |  |  | 6029 | -0.076 | 0.0366 | No |
| 281 | NR3C1 |  |  | 6073 | -0.078 | 0.0326 | No |
| 282 | C1orf198 |  |  | 6097 | -0.079 | 0.0309 | No |
| 283 | ELK3 |  |  | 6113 | -0.080 | 0.0301 | No |
| 284 | ATP6V0E1 |  |  | 6138 | -0.082 | 0.0283 | No |
| 285 | LASP1 |  |  | 6267 | -0.089 | 0.0145 | No |
| 286 | DCAF6 |  |  | 6327 | -0.091 | 0.0088 | No |
| 287 | SHROOM3 |  |  | 6397 | -0.095 | 0.0019 | No |
| 288 | IQGAP2 |  |  | 6450 | -0.099 | -0.0029 | No |
| 289 | PYGL |  |  | 6492 | -0.101 | -0.0064 | No |
| 290 | TJP1 |  |  | 6500 | -0.101 | -0.0060 | No |
| 291 | UGDH |  |  | 6647 | -0.111 | -0.0216 | No |
| 292 | LIX1L |  |  | 6656 | -0.111 | -0.0212 | No |
| 293 | ENAH |  |  | 6658 | -0.111 | -0.0200 | No |
| 294 | NBR1 |  |  | 6667 | -0.112 | -0.0195 | No |
| 295 | ANXA6 |  |  | 6668 | -0.112 | -0.0182 | No |
| 296 | CSRP1 |  |  | 6689 | -0.113 | -0.0191 | No |
| 297 | RBMS1 |  |  | 6694 | -0.114 | -0.0182 | No |
| 298 | PTPN14 |  |  | 6704 | -0.114 | -0.0179 | No |
| 299 | EHD2 |  |  | 6714 | -0.114 | -0.0175 | No |
| 300 | FLNA |  |  | 6856 | -0.126 | -0.0324 | No |
| 301 | PXDC1 |  |  | 6879 | -0.128 | -0.0334 | No |
| 302 | MOB1A |  |  | 6892 | -0.128 | -0.0332 | No |
| 303 | JAK1 |  |  | 6893 | -0.128 | -0.0316 | No |
| 304 | ARL4A |  |  | 6930 | -0.131 | -0.0342 | No |
| 305 | BAG3 |  |  | 6933 | -0.132 | -0.0328 | No |
| 306 | KANK2 |  |  | 6981 | -0.135 | -0.0367 | No |
| 307 | PLS3 |  |  | 7000 | -0.137 | -0.0371 | No |
| 308 | SYDE1 |  |  | 7021 | -0.139 | -0.0377 | No |
| 309 | ARL1 |  |  | 7036 | -0.140 | -0.0376 | No |
| 310 | PRRX1 |  |  | 7057 | -0.142 | -0.0382 | No |
| 311 | PLK2 |  |  | 7069 | -0.143 | -0.0378 | No |
| 312 | ZCCHC24 |  |  | 7078 | -0.143 | -0.0370 | No |
| 313 | TMEM263 |  |  | 7170 | -0.153 | -0.0457 | No |
| 314 | RGS3 |  |  | 7193 | -0.155 | -0.0463 | No |
| 315 | HIBADH |  |  | 7220 | -0.158 | -0.0474 | No |
| 316 | SDCBP |  |  | 7222 | -0.158 | -0.0456 | No |
| 317 | DUSP6 |  |  | 7258 | -0.162 | -0.0477 | No |
| 318 | RGS10 |  |  | 7263 | -0.163 | -0.0462 | No |
| 319 | MYL12A |  |  | 7289 | -0.166 | -0.0471 | No |
| 320 | PHLDA3 |  |  | 7307 | -0.168 | -0.0470 | No |
| 321 | UAP1 |  |  | 7314 | -0.168 | -0.0457 | No |
| 322 | CTDSP2 |  |  | 7391 | -0.180 | -0.0523 | No |
| 323 | TUBB6 |  |  | 7396 | -0.180 | -0.0506 | No |
| 324 | RNH1 |  |  | 7414 | -0.183 | -0.0503 | No |
| 325 | ZFP36L1 |  |  | 7466 | -0.188 | -0.0540 | No |
| 326 | DUSP14 |  |  | 7487 | -0.190 | -0.0540 | No |
| 327 | DESI2 |  |  | 7500 | -0.191 | -0.0531 | No |
| 328 | ACTN1 |  |  | 7520 | -0.193 | -0.0529 | No |
| 329 | FAM120A |  |  | 7521 | -0.193 | -0.0506 | No |
| 330 | TGFB1I1 |  |  | 7559 | -0.199 | -0.0525 | No |
| 331 | GSN |  |  | 7578 | -0.201 | -0.0521 | No |
| 332 | PDLIM1 |  |  | 7628 | -0.208 | -0.0553 | No |
| 333 | CLIC4 |  |  | 7637 | -0.209 | -0.0537 | No |
| 334 | NANS |  |  | 7653 | -0.211 | -0.0528 | No |
| 335 | SEC14L1 |  |  | 7736 | -0.223 | -0.0596 | No |
| 336 | CMTM3 |  |  | 7797 | -0.234 | -0.0638 | No |
| 337 | ID1 |  |  | 7821 | -0.238 | -0.0635 | No |
| 338 | EPS8 |  |  | 7879 | -0.250 | -0.0671 | No |
| 339 | RAP1A |  |  | 7907 | -0.257 | -0.0671 | No |
| 340 | ARPC1B |  |  | 7917 | -0.259 | -0.0650 | No |
| 341 | ABRACL |  |  | 7955 | -0.266 | -0.0661 | No |
| 342 | CETN2 |  |  | 7970 | -0.270 | -0.0644 | No |
| 343 | TPM1 |  |  | 8012 | -0.278 | -0.0658 | No |
| 344 | MBD2 |  |  | 8013 | -0.278 | -0.0625 | No |
| 345 | SQSTM1 |  |  | 8044 | -0.284 | -0.0625 | No |
| 346 | CTNNA1 |  |  | 8047 | -0.284 | -0.0593 | No |
| 347 | CAPN2 |  |  | 8080 | -0.293 | -0.0594 | No |
| 348 | FAM114A1 |  |  | 8104 | -0.298 | -0.0585 | No |
| 349 | RAP1B |  |  | 8181 | -0.320 | -0.0634 | No |
| 350 | PALLD |  |  | 8288 | -0.351 | -0.0714 | No |
| 351 | NFIC |  |  | 8297 | -0.354 | -0.0681 | No |
| 352 | LITAF |  |  | 8324 | -0.361 | -0.0667 | No |
| 353 | SOX9 |  |  | 8327 | -0.361 | -0.0625 | No |
| 354 | GNG12 |  |  | 8328 | -0.362 | -0.0581 | No |
| 355 | WWTR1 |  |  | 8384 | -0.383 | -0.0599 | No |
| 356 | SH3BGRL |  |  | 8409 | -0.390 | -0.0579 | No |
| 357 | LMNA |  |  | 8478 | -0.423 | -0.0607 | No |
| 358 | PEA15 |  |  | 8509 | -0.439 | -0.0588 | No |
| 359 | HSPB1 |  |  | 8586 | -0.478 | -0.0618 | No |
| 360 | NQO1 |  |  | 8588 | -0.479 | -0.0561 | No |
| 361 | RAB13 |  |  | 8590 | -0.481 | -0.0504 | No |
| 362 | ANXA1 |  |  | 8595 | -0.484 | -0.0449 | No |
| 363 | ANXA5 |  |  | 8626 | -0.510 | -0.0422 | No |
| 364 | VIM |  |  | 8663 | -0.535 | -0.0399 | No |
| 365 | PRDX6 |  |  | 8673 | -0.540 | -0.0344 | No |
| 366 | ID3 |  |  | 8676 | -0.544 | -0.0280 | No |
| 367 | POLR2L |  |  | 8715 | -0.578 | -0.0254 | No |
| 368 | CNN3 |  |  | 8752 | -0.613 | -0.0221 | No |
| 369 | RHOC |  |  | 8823 | -0.676 | -0.0220 | No |
| 370 | RAB31 |  |  | 8831 | -0.687 | -0.0145 | No |
| 371 | ANXA2 |  |  | 8868 | -0.729 | -0.0098 | No |
| 372 | MYL12B |  |  | 8891 | -0.760 | -0.0031 | No |
| 373 | LGALS1 |  |  | 8982 | -1.135 | 0.0002 | No |
Table: GSEA details [plain text format]

  

Fig 2: VANGRONINGEN\_MES: Random ES distribution      
 Gene set null distribution of ES for **VANGRONINGEN\_MES**
